# The Emerging Roles of Tripartite Motif Proteins (TRIMs) in Acute Lung Injury

**DOI:** 10.1155/2021/1007126

**Published:** 2021-10-19

**Authors:** Yingjie Huang, Yue Xiao, Xuekang Zhang, Xuan Huang, Yong Li

**Affiliations:** ^1^Department of Anesthesiology, The First Affiliated Hospital of Nanchang University, Nanchang, China; ^2^The First Clinical Medical College, Nanchang University, Nanchang 330006, China; ^3^The National Engineering Research Center for Bioengineering Drugs and the Technologies, Institute of Translational Medicine, Nanchang University, Nanchang, China

## Abstract

Acute lung injury (ALI) is an inflammatory disorder of the lung that causes high mortality and lacks any pharmacological intervention. Ubiquitination plays a critical role in the pathogenesis of ALI as it regulates the alveolocapillary barrier and the inflammatory response. Tripartite motif (TRIM) proteins are one of the subfamilies of the RING-type E3 ubiquitin ligases, which contains more than 80 distinct members in humans involved in a broad range of biological processes including antivirus innate immunity, development, and tumorigenesis. Recently, some studies have shown that several members of TRIM family proteins play important regulatory roles in inflammation and ALI. Herein, we integrate emerging evidence regarding the roles of TRIMs in ALI. Articles were selected from the searches of PubMed database that had the terms “acute lung injury,” “ubiquitin ligases,” “tripartite motif protein,” “inflammation,” and “ubiquitination” using both MeSH terms and keywords. Better understanding of these mechanisms may ultimately lead to novel therapeutic approaches by targeting TRIMs for ALI treatment.

## 1. Introduction

Acute lung injury (ALI) is an acute hypoxic respiratory insufficiency caused by various direct (pulmonary) or indirect (extrapulmonary) injuries including sepsis syndrome, ischemia-reperfusion, pneumonia, and mechanical ventilation, which leads to the destruction of the barrier of alveolar epithelial cells and capillary endothelial cells, resulting in overinfiltration of inflammatory cells and diffuse pulmonary interstitial and alveolar edema [1]. In 1994, the diagnostic criteria of ALI were put forward by the American–European Consensus Conference: an acute onset; oxygenation index (PaO_2_/FiO_2_) > 200 mm Hg and < 300 mm Hg (1 mmHg = 0.133 kPa); patchy shadows in both lungs on the chest X-ray; pulmonary artery wedge pressure ≤ 18 mmHg or no clinical evidence of left atrial hypertension; etc. [2]. Due to the lack of drug intervention, ALI remains a significant cause of morbidity and mortality in the critically ill patient population [2]. More severe situations with PaO_2_/FiO_2_ ≤ 200 mm Hg, ALI turns to the worse stage acute respiratory distress syndrome (ARDS) [3].

Pathological hallmarks of ALI are injury to the vascular endothelium/alveolar epithelium, activation of innate immune response, and enhanced coagulation [4]. Exposure to several risk factors (i.e., pneumonia, sepsis, and shock) firstly leads to endothelial and/or epithelial monolayers damage, increasing permeability and impairing their barrier function [4]. A large amount of protein-rich fluid and inflammatory cells leaks into the alveoli and lung interstitium, resulting in pulmonary edema, neutrophil infiltration, cytokine and reactive oxygen species-mediated inflammation coagulation disorders, and pulmonary fibrosis [5]. Notedly, mast cells (MCs) and polymorphonuclear neutrophils (PMNs) are the main inflammatory cells, which play a critical role in the pathogenesis of ALI [6, 7]. MC activation induces tryptase release to trigger ALI [8], which is supported by the finding that MCs “stabilizers” can reduce ALI severity [9].

At present, there is still no effective pharmaceutical intervention for ALI; mechanical ventilation is the main approach to prevent respiratory failure and, combined with intensive care support, could improve health condition [10]. However, mechanical ventilation can exacerbate preexisting lung injury or even induce de novo injury in healthy lungs, which is called ventilator-induced lung injury (VILI) [11, 12]. In recent years, researchers have paid close attention to the identification of new routes at cellular level which could provide a better understanding of the physiopathology of ALI, but the precise cellular and molecular underlying mechanisms are still to be fully elucidated. Chen et al. recently summarized and introduced the role of lncRNAs in ALI in detail [13]. However, emerging evidence points out to the ubiquitination which functions as an important regulator in the pathobiology of ALI since it regulates the proteins evolved in the modulation of the alveolocapillary barrier and inflammatory response, opening a highly promising research field for the treatment of lung diseases [14].

## 2. Ubiquitination in ALI

Ubiquitination is the major protein posttranslational modification in cells by which ubiquitin (Ub) covalently attached the target protein for degradation through the 26S proteasome or lysosome or nonproteolytic modifications [15]. It plays crucial roles in diverse biological processes such as DNA repair, cell proliferation, signal transduction, apoptosis, and inflammation, whose dysregulation leads to many diseases [16]. However, bacterial infection or inflammatory stimulation often disrupts the process of protein ubiquitination. We and some other investigators have shown that expressions of some E3 Ub ligases were altered by infection or inflammation thus affecting the levels and functions of their target proteins. Thus, uncovering new E3 Ub ligase-related molecular mechanisms and signaling pathways will provide a unique opportunity for the potential design of new strategies to alleviate ALI.

### 2.1. The Ubiquitin System

As a multicomponent regulatory system, the Ub system is composed of three types of Ub enzymes, which is a highly controlled mechanism of protein degradation and turnover in cells, starting with approximately 8 kDa monomeric Ub [17]. Ub is activated by a Ub-activating enzyme (E1) in an adenosine-triphosphate- (ATP-) dependent manner and then conjugated by a ubiquitin conjugating enzyme (E2), finally resulting in transfer of Ub to an internal lysine of the substrate protein by an E3 ligase [18]. To date, there are only two E1 enzymes (UBA1 and UBA6) [19], around 40 E2 enzymes [20], but more than 600 E3 ligases [21] existed in the human genome. Although the addition of Ub moieties to specific residues on a substrate protein is partly because of E2/E3 enzymes pairings, E3 ligases were considered the predominant source of substrate specificity [22].

### 2.2. E3 Ligases

As a direct mediator of substrate tagging and ubiquitin chain elongation, E3 ligase is considered an essential component in the ubiquitin system that determines substrate specificity. In human genome, more than 600 putative E3 ligases have been identified [21]. There are three major kinds of E3 ligases divided by the molecular structure and functional mechanism, including HECT (homologous to the E6-associated protein carboxyl-terminus) domain family, RING (really interesting new gene) finger family, and the RBR (RING in-between-RING) E3 ubiquitin ligases [23]. HECT E3 ligase contains an N-terminal lobe which is responsible for E2 binding and substrate recognition, and a C-terminal HECT domain containing a catalytic cysteine that receives and passes an ubiquitin molecule from the E2 enzyme before conjugating ubiquitin to a substrate protein [24]. RING finger E3 ligases constitute the largest family of E3 ligases which are characterized based on the presence of a RING domain [25]. Interestingly, the canonical RING domain is a type of zinc finger with a RING fold structure while another type is the U-box domain which possesses the same RING fold but without zinc [26]. Unlike HECT E3 ligase, RING finger E3 ligase mediates the direct transfer of ubiquitin to a substrate protein by binding to a ubiquitin-charging E2 enzyme as a scaffold [27]. Notably, RING E3 ligases function either as monomers (e.g., c-CBL, E4B), homodimers (e.g., cIAP, CHIP), heterodimers (e.g., Mdm2-MdmX), or large multisubunit complexes, such as the Cullin-RING ligases (CRLs), which make up a distinct subtype characterized by their common Cullin scaffold protein [23]. RBR E3 ligases contain two RING domains (RING1 and RING2) with an in-between-RING (IBR) domain and share the common features of both HECT and RING finger E3 ligases which function as a hybrid of these two types of E3 ligases [28]. Specifically, the RING1 domain binds to ubiquitin-loaded E2 and transfers ubiquitin onto the RING2 domain at a catalytic cysteine residue before conjugation to the substrate protein [29].

### 2.3. Role of E3 Ligases in ALI

Although ubiquitination has been reported to play a pivotal role in multiple biological functions, its function in ALI remains poorly understood. Recently, the key regulative role of ubiquitination in ALI has been mentioned increasingly [14]. Of them, most studies have been focused on the E3 ubiquitin ligases and the conventional K48-ubiquitination which leads to the substrate proteins degradation via the 26S proteasome [30]. Accumulating evidence has demonstrated that E3 ligase plays a critical role in the pathobiology of ALI since it modulates critical proteins involved in the alveolocapillary barrier and the inflammatory response [14].

Tight junctions form a highly selective diffusion barrier between endothelial cells and epithelial cells by preventing most dissolved molecules and ions from passing freely through the paracellular pathway [31]. The function impairment of tight junction is a sign of ALI [32]. E3 ligase Itch, a member of the HECT Ub ligases, could directly interact with and degrade the tight junction-specific protein occludin [33] via ubiquitination. E-cadherin, a well-studied member of the classical cadherin family, is a central component in the cell-cell adhesion junction and plays a critical role in maintaining cell polarity and the integrity of epithelial cells [34]. Dysfunction of E-cadherin contributes to the pathogenesis of ALI [35]. A RING finger E3 ligase Hakai induces E2-dependent ubiquitination and endocytosis of E-cadherin complex in epithelial cells [36]. Recently, Dong et al. found that the HECT E3 ligase Smurf2 induced *μ*-opioid receptor 1 (MOR1) degradation in the ubiquitin-proteasome system in lung epithelial cells, and MOR1 has a potential effect in lung repair and remodeling after ALI [37].

E3 ligase Cblb inhibits the MyD88-dependent Toll-like receptor 4 (TLR4) signaling and attenuates acute lung inflammation induced by polymicrobial sepsis [38]. The ST2L receptor for interleukin 33 (IL-33) mediates pulmonary inflammation during ALI, which is bound and ubiquitinated by FBXL19, a member of the Skp1-Cullin-F-box family of E3 ubiquitin ligases [39]. In addition, E3 ligase FBXO3 targets the TRAF inhibitor FBXL2 for its destabilization and potently stimulates cytokine release, leading to changes in lung permeability, alveolar edema, and ALI [40]. FBXO17 has been described as an E3 ligase that recognizes and mediates the ubiquitination and degradation of GSK3*β* to reduce inflammatory responses in lung epithelial cells after LPS injury [41]. Most recently, Lear et al. reported that E3 ligase KIAA0317 targets SOCS2 for ubiquitination and degradation by the proteasome and exacerbates pulmonary inflammation [42]. These studies have proved that ubiquitin-proteasome system especially E3 ligases is closely related to the pathogenesis of lung injury.

## 3. TRIMs in ALI

TRIM proteins are regarded as a subfamily of the RING finger E3 ligase, which contain more than 80 distinct members in humans [43]. TRIMs are composed by conserved three zinc-binding domains, an N-terminal RING domain, one or two B-boxes, and a central coiled-coiled domain (CDD) [44]. We recently found that TRIM65 selectively targeted vascular cell adhesion molecule 1 (VCAM-1) and promoted its ubiquitination and degradation, by which it critically controlled the duration and magnitude of pulmonary inflammation in ALI [45]. Particularly, Whitson and his colleagues reported that TRIM72 (also known as MG53) could function as a novel therapeutic protein to treat ALI [46]. Here, we discuss our current understanding of TRIMs as E3 ligases that executes its effector functions in ALI (Table 1).

### 3.1. TRIM8

TRIM8, as a member of TRIM family, has a common structural feature of a typical RBCC motif as well as a monopartite nuclear localization signal (NLS), which allows shuttling and functioning into the nucleus [47]. It has been reported that TRIM8 can regulate NF-*κ*B signaling both in the nucleus and cytoplasm: TRIM8 inhibited PIAS3-mediated negative regulation of p65 to enhance NF-*κ*B activity in the nucleus [48] and can also positively regulate NF-*κ*B pathway through k63-linked polyubiquitination of cytoplasmic protein TAK1 [49]. TRIM8 is ubiquitously expressed in human and mouse tissues, which has higher expression levels in the central nervous system, kidney, and lens, and lower expression level in digestive tract [44]. TRIM8 plays a key role in the immune response and participates in various fundamental biological processes such as cell survival, apoptosis, autophagy, differentiation, inflammation, and carcinogenesis [50].

Recently, studies have revealed that TRIM8 is involved in the regulation of sepsis and ALI. TRIM8 was significantly upregulated in LPS (lipopolysaccharide) sepsis-induced acute hepatic injury (AHI), which was a direct target of miR-373-3p [51]. Moreover, inhibition of TRIM8 by downregulation of long noncoding RNA (lncRNA) LINC00472, which served as a sponge for miR-373-3p and negatively regulated its expression, could reduce sepsis-induced expression of main proinflammatory cytokines such as IL-6, IL-10, and TNF-*α* [51]. Xiaoli et al. found that TRIM8 was increased in a time-dependent manner during LPS-induced ALI, promoting inflammatory response and ROS generation via the inactivation of p-AMPK*α*. In addition, suppression of TRIM8 markedly downregulated mRNA levels of interleukin-1*β* (IL-1*β*), IL-6, and tumor necrosis factor-*α* (TNF-*α*) in lung epithelial cells mainly through blocking the NF-*κ*B signaling pathway and alleviated oxidative stress by regulating Nrf2 signaling and heme oxygenase-1 (HO-1) expression [52]. Although TRIM8 has been revealed to play an important role in acute lung injury, precise regulatory mechanisms such as whether it depends on the activity of E3 ubiquitin ligase and its specific target proteins need to be further clarified.

### 3.2. TRIM14

TRIM14 was originally known as KIAA0129 [53], and its overexpression was first found in human immunodeficiency virus- (HIV-) infected human and simian non-Hodgkin's lymphoma infected with simian immunodeficiency virus (SIV) [54, 55]. TRIM14 is a noncanonical member of the TRIM family, since it lacks the N-terminal RING domain of the typical RBCC motif which can exert an E3 ubiquitin ligase [56]. Studies have shown that TRIM14 can perform various functions via partners with which directly interact with its PRYSPRY domain [57]. Interestingly, TRIM14 was reported to be an important mediator of antiviral immunity both in DNA virus and double-stranded RNA virus infection [58, 59]. Furthermore, several groups found that TRIM14 may be involved in tumorigenesis [60–64].

Recently, we found that TRIM14 was overexpressed in human vascular endothelial cells (ECs) and markedly induced by inflammatory stimuli such as LPS [65]. TRIM14 was a new positive regulator of endothelial activation via activating the NF-*κ*B signaling pathway, which can directly bind to the promoter of *TRIM14* gene and control its transcription [65]. Zhou et al. revealed that TRIM14 underwent Lys-63-linked autopolyubiquitination at Lys-365 and served as a platform and recruited NEMO to the mitochondrial antiviral signal (MAVS) complex, leading to the activation of interferon regulatory factor 3 (IRF3) and NF-*κ*B signaling in human lung epithelial cells, which boost antiviral innate immune response [66]. TRIM14 also can recruit USP14 to cleave the K63-linked ubiquitin chain at lysine 332/338/341 of p100/p52, hinder the recognition of receptor p62, and inhibit the autophagy degradation of p100/p52, thus promoting the atypical activation of NF-*κ*B *in vivo* and *in vitro* [67]. Considering endothelial inflammation and dysfunction play a prominent role in development of ALI and NF-*κ*B is a central transcriptional factor in ALI, it is suggested that TRIM14 may be involved in the pathological process of ALI, which needs further study.

### 3.3. TRIM21

TRIM21, also known as Ro52, has a typical RBCC motif and E3 ligase activity [68]. It is broadly expressed in most human tissues and cells and predominantly expressed in hematopoietic cells and endothelial and epithelial cells [69]. TRIM21 was identified as a major autoantigen in autoimmune diseases including Sjögren's syndrome, systemic lupus erythematosus (SLE), and rheumatoid arthritis [70–72]. Later studies revealed that TRIM21 is a highly conserved IgG receptor with high cytoplasmic affinity and specificity [73, 74], which can be induced by interferon to exert antiviral effect [75]. TRIM21 serves as a multifaceted regulator in viral immunity and can not only promote the production of type I interferon [76] and triggers an innate immune response via RIG-1 and cGAS sensing [77] but also negatively regulate innate immunity by targeting and degrading the viral DNA sensor DEAD (Asp-Glu-Ala-Asp) box polypeptide 41 (DDX41) [78]. The biological function and application of TRIM21 in antiviral immunity are described in detail in other reviews [79].

Using TRIM21-deficient mice, Yoshimi and colleagues found that TRIM21 is a negative regulator of NF-*κ*B-dependent proinflammatory cytokine production induction in fibroblasts after TLR ligands (poly(I:C), CpG, and LPS) stimulation [80]. In addition, TRIM21 deletion can lead enhanced production of proinflammatory cytokines and systemic autoimmunity though the IL-23-Th17 pathway [81]. Recently, Li et al. reported that TRIM21 exhibited an anti-inflammatory property against LPS-induced lung endothelial dysfunction and monocytes adhesion to endothelial cells [82]. TRIM21 can be monoubiquitylated and lysosomal degraded in response to LPS and may contribute to the pathogenesis of ALI [52]. TRIM21 can be used as a therapeutic target for endothelial dysfunction induced by sepsis, such as acute lung injury [83]. However, whether ubiquitination of TRIM21 is dependent on its phosphorylation and the specific phosphorylation or ubiquitination sites needs to be further clarified.

### 3.4. TRIM65

Human TRIM65 is a 517-amino acid protein, containing a N-terminal RING domain, a B-box, a coiled-coil domain, and a SPRY domain, is first known as a gene associated with white matter lesions [84, 85]. Using a systematic discovery-type proteomic analysis, Li et al. found that TRIM65 can negatively regulate miRNA-mediated mRNA translation inhibition through ubiquitination and subsequent degradation of trinucleotide repeat containing six (TNRC6) [86, 87]. Like other TRIMs, TRIM65 also participates in the antiviral innate immune response by ubiquitination of MDA5 [88, 89]. Over the years, several reports suggest that TRIM65 acts as a ubiquitin E3 ligase, targeting p53, ANXA2, Axin1, and ARHGAP35 to regulate carcinogenesis [90–93]. Most recently, Liu et al. published a review to introduce TRIM65 in white matter lesions, innate immunity and tumor [94].

We recently found that TRIM65 may control the magnitude and duration of LPS-induced lung inflammation and injury [45]. TRIM65-deficient (TRIM65^−/−^) mice are more sensitive to LPS-induced death due to sustained and severe pulmonary inflammation. Further studies showed that monocytes/macrophages were higher in the BAL from TRIM65^−/−^ mice, by which TRIM65 selectively targets vascular cell adhesion molecule 1 (VCAM-1) and directly induces its ubiquitination degradation in endothelial cells. It is worth noting that TRIM65 does not affect the MAPK and NF-*κ*B signaling pathways in ALI, although some studies have revealed that TRIM65 can activate the Erk1/2 pathway [95, 96], which suggests that TRIM65 has diverse functions in different cells and under distinct pathological conditions. Furthermore, TRIM65 is enriched in endothelial cells and declined at the early stage during endothelial activation; the mechanisms that precisely regulate TRIM65 levels in endothelial inflammation remain unknown. Further studies are necessary to understand the regulatory mechanisms that control TRIM65 expression.

### 3.5. TRIM72

TRIM72 (also known as MG53) is composed of a typical TRIM family protein RBCC structure and a PRY-SPRY subdomain which is mainly expressed in cardiac and skeletal muscle, as well as in renal and alveolar epithelial cells, monocyte, and macrophages with detectable amount level [97–100]. Cai et al. first revealed that TRIM72 acted as a key component of the sarcolemma cell plasma membrane repair machinery [101]. Upon the membrane injurious stimuli, TRIM72 oligomerized by oxidizing the thiol group of cysteine at position 242 and a leucine zipper motif to induce the intracellular vesicles coated with TRIM72 to nucleate at the injured site, resulting in resealing the damaged membrane [102, 103]. At the membrane, TRIM72 protein binds to phosphatidyl serine to mediate the recruitment of vesicles at the injured site [104]. Interestingly, TRIM72 can be secreted and circulate throughout the entire body to reach all tissues and organs, allowing the recombinant TRIM72 protein to have therapeutic benefit in treatment of injuries to multiple tissues, such as the heart, kidney, lung, brain, liver, skin, skeletal muscle, and cornea [105].

Ablation of the TRIM72 gene leads to increased susceptibility to ischemia-reperfusion and overventilation-induced ALI in mice [97]. Recently, Sermersheim and his colleagues found that knockdown of TRIM72 in macrophages results in activation of NF-*κ*B signaling and increased inflammatory factor interleukin-1*β* upon influenza virus infection, and knockout of TRIM72 promotes CD45^+^ cells infiltration and IFN*β* elevation in the lung [98]. Kenney et al. found that exogenous injection of recombinant human TRIM72 protein could protect ALI caused by lethal influenza virus infection [106]. Recombinant TRIM72 protein significantly decreased the level of inflammatory cytokines of IFN*β*, IL-6, and IL-1*β* and infiltrating CD11b^+^ lymphocytes in lung tissues [106]. It is reported that intravenous (IV) delivery or inhalation of recombinant human TRIM72 protein reduces symptoms in rodent models of ALI and emphysema [97]. The extracellular recombinant protein protects cultured lung epithelial cells against anoxia/reoxygenation-induced injuries [97]. Most recently, Whitson et al. had evaluated the therapeutic benefits of recombinant human TRIM72 protein in porcine models of ALI and found that recombinant TRIM72 protein can mitigate lung injury in the porcine model of combined hemorrhagic shock/contusive lung injury and reduce warm ischemia-induced injury to the isolated porcine lung through ex vivo lung perfusion administration [46]. These findings revealed that TRIM72 plays a critical role in ALI, and exogenous-recombinant TRIM72 protein may be a shelf stable therapeutic agent with the potential to restore lung function and lessen the impact of ALI.

## 4. Conclusions

TRIMs are a wide and well-conserved family of proteins defined as a subfamily of the RING-type E3 Ub ligases, which have been implicated in a broad range of biological processes including antiviral immunity, cell differentiation, development, and carcinogenesis. Accumulating evidence has shown that several TRIM members have unique and vital roles in ALI using distinct mechanisms (Table 1). Particularly, the regulation of ALI by targeting cell membrane repair has been a focus of intense research in the last few years. Interestingly, systemically administered recombinant human TRIM72 proteins could recognize injury to both epithelial and endothelial layers in the lung, which can effectively preserve lung structure and function in ALI. TRIM72 will be one of the most promising therapeutic agents with the potential to restore lung function and lessen the impact of ALI. Further work is needed to understand full contribution of TRIMs including discovered and undiscovered members to ALI. Identification of TRIM proteins with the potential to serve as therapeutic targets of ALI may help to novel drug development of ALI treatment.

## Figures and Tables

**Table 1:** Role of TRIMs in acute lung inflammation.

TRIMs	Models	Cell types	Mechanisms	Ref No.
TRIM8	LPS-induced AHI	Human liver cells	LINC00472/miR-373-3p/TRIM8 axis	[51]
	LPS-induced ALI	Lung epithelial cells	p-AMPK*α*/NF-*κ*B/Nrf2/ROS/HO-1 axis	[52]
TRIM14	LPS-induced ALI	Human vascular endothelial cells	NEMO/TAK1/NF-*κ*b/TRIM14 pathway	[63]
TRIM21	LPS-induced ALI	Lung microvascular endothelial cells	NF-*κ*B signaling	[79]
TRIM65	ALI	Human vascular endothelial cells	VACM-1 ubiquitination and degradation	[45]
TRIM72	Ischaemia-reperfusion and overventilation-induced ALI	Lung epithelial cells	Cell membrane repair	[97]
	Influenza virus -induced ALI	Macrophage Lung tissue	NF-*κ*B signaling Inhibition of pyroptosis	[98, 106]
	Hemorrhagic shock/contusive ALI	Human bronchial epithelial cells	Cell membrane repair	[46]

## Data Availability

No data were used to support this study.
